# The Biology of Glial Cells and Their Complex Roles in Alzheimer’s Disease: New Opportunities in Therapy

**DOI:** 10.3390/biom8030093

**Published:** 2018-09-10

**Authors:** Saif Shahriar Rahman Nirzhor, Rubayat Islam Khan, Sharmind Neelotpol

**Affiliations:** Department of Pharmacy, BRAC University, Dhaka 1212, Bangladesh; rubayat.khan@bracu.ac.bd (R.I.K.); sharmind@bracu.ac.bd (S.N.)

**Keywords:** glial cells, astrocytes, NG2 glia, microglia, oligodendrocytes, Alzheimer’s disease, neurodegenerative disease, Aβ-peptides

## Abstract

Even though Alzheimer’s disease (AD) is of significant interest to the scientific community, its pathogenesis is very complicated and not well-understood. A great deal of progress has been made in AD research recently and with the advent of these new insights more therapeutic benefits may be identified that could help patients around the world. Much of the research in AD thus far has been very neuron-oriented; however, recent studies suggest that glial cells, i.e., microglia, astrocytes, oligodendrocytes, and oligodendrocyte progenitor cells (NG2 glia), are linked to the pathogenesis of AD and may offer several potential therapeutic targets against AD. In addition to a number of other functions, glial cells are responsible for maintaining homeostasis (i.e., concentration of ions, neurotransmitters, etc.) within the central nervous system (CNS) and are crucial to the structural integrity of neurons. This review explores the: (i) role of glial cells in AD pathogenesis; (ii) complex functionalities of the components involved; and (iii) potential therapeutic targets that could eventually lead to a better quality of life for AD patients.

## 1. Introduction

A debilitating neurological disorder, Alzheimer’s disease (AD) is characterized by β-amyloid (Aβ) induced senile plaques and hyper-phosphorylated tau protein aggregation in the brain leading to a loss in cognitive ability in patients and eventually dementia [1]. With age being one of its greatest risk factors, AD is being increasingly observed in older individuals throughout the world. The approximate number of patients with AD in 2010 was 35 million and this number is projected to rise to 65 million in 2030. The prevalence of AD is almost double in human males compared to females and after the age of 85 the occurrence of AD is seen in almost 50% of males [1]. Alzheimer’s disease can be classified into two different subgroups based on the age of onset of the disease. Familial AD (FAD) occurs at an early age of onset and Sporadic AD (SAD) occurs at a much later age. Familial AD is a rare autosomal dominant genetic disorder that accounts for only a minor portion of AD cases. Familial AD symptoms appear in patients between the age of 30 and 50; and this specific disorder is characterized by mutations in amyloid precursor protein or presenilin 1 and 2 genes as well as tau protein hyperphosphorylation. SAD accounts for the majority of AD cases and the usual age of onset is observed to be 65 onwards. Even though the underlying mechanism of SAD remains unknown, SAD has been linked to the Apolipoprotein E (ApoE) gene [2,3]. There is significant evidence to support that a major mechanism by which ApoE influences AD is through ApoE induced Aβ peptide deposition. This occurs in both a dose-specific and isoform-specific fashion, i.e., APOE4  >  APOE3  >  APOE2. It has been seen that APOE4 is the most prominent risk factor for late-onset AD and ApoE polymorphism and ApoE allele, not necessarily mutations, are thought to be a risk factor for AD progression. APOE4 mice displayed increased glial activation in response to lipopolysaccharides (LPS) compared to APOE2 and APOE3 transgenic mice further elucidating the fact that ApoE polymorphism is mainly modulating AD progression to some extent. However, in addition to ApoE, there are other mechanisms that may be in play here too [4,5,6,7]. There are several methods of assessment available for AD patients, including tests of episodic memory and attention span, but a more definite diagnosis of AD can only be made by post-mortem histological analysis. These histological analyses reveal cerebral cortical atrophy, amyloid plaques, neurofibrillary tangles (NFTs) and vascular amyloidosis that collectively represent Aβ peptide deposits in the brain [8,9]. Much of the research on AD thus far has been very neuron-oriented but, recently, the interest in glial cells in this particular topic is markedly increasing.

### 1.1. Overview of the Role of Glial Cells in Alzheimer’s Disease

The nervous system is built with two types of cells, i.e., neurons and glial cells. The actual number of glial cells in comparison to neurons has been an unanswered question for a number of years but recent cell counting methods such as the isotropic fractionator suggest that glial cells may actually be smaller in number [10]. The glial cells involved in the nervous system are: oligodendrocytes, polydendrocytes (NG2 glia), astrocytes, Schwann cells, satellite cells, microglia, ependymal cells and enteric glial cells. These glial cells function to maintain homeostasis (i.e., concentration of ions, neurotransmitters, etc.) within the neuronal vicinity, aid in the formation of myelin sheaths around the axons in the nervous system responsible for the cell-to-cell signaling and maintain the function of synapses [11]. Homeostatic disruption may accelerate the progression of neurodegenerative diseases such as AD [11]. Recent studies limn the importance of glial cells in AD research through in vivo studies in variegated animal models.

### 1.2. In Vivo Models Used for the Study of Alzheimer’s Disease

Animal models, especially murine models have been widely used in the studies regarding AD pathogenesis. Sporadic Alzherimer’s Disease (SAD) is the more common form of AD that is difficult to study compared to FAD; hence, SAD animal models are non-existent. However, studying FAD is somewhat more feasible to observe in murine models. If there is an underlying assumption that the two of types of AD have similar genetic origin, then AD may be studied more practically using these models. Some murine studies that have been pivotal in identifying the neuropathology of AD [12,13,14,15,16,17,18,19,20,21] studies on specific types of glial cells have been done on specific mouse models. Table 1 summarizes some well-studied murine models for AD and its corresponding alterations in specific types of glial cells. These are just a few examples and a comprehensive guide to these murine models and relevant findings can be found on the Alzforum website (https://www.alzforum.org/research-models) where a database is maintained to aid in AD research.

These animal models are indeed helpful in terms of looking at the pathogenesis of AD; however, it is worth noting that these models do not accurately mimic AD and the many complexities it creates. Moreover, there could be several problems related to the interpretation of these kinds of data from animal models to humans that may obfuscate the lucidity of the observed phenomena. For example, in some of these models, fragments of Amyloid precursor protein (APP), such as intracellular cytoplasmic domain (AICD) or α-secretase cleaved secreted APP (α-APPα), may also show overexpression along with APP. These fragments may exhibit some functions in the host that may be observable clinically. For example, in Tg2576 mice (Table 1), the overexpressed α-APPα upregulates insulin like growth factor-2 (IGF-2) and protein transthyretin (TTR) that may lead to some neuroprotective properties for the mice. This may be one of the reasons these mice often do not show a significant amount of neuronal loss [22]. Another reason the interpretation may be hard is that wild-type mice that overexpress APP may show cognitive decline even without Aβ deposits being formed in the brain [23]. The interpretation becomes even more nebulous for a modeled organism when, due to alternative splicing, the transgene mRNA of APP might be altered and the composition may differ from endogenous APP. This becomes even less manageable when the variability across different organisms is taken into account. This variability is an issue since the models are usually selected by looking at the phenotype similarity between the model and human [24,25]. Regardless of the challenges, murine models have been a useful tool in identifying the roles of various glial cells in the pathogenesis of AD and this review focuses on these individual cell types and their potential therapeutic applications separately.

## 2. Microglia

Microglia is an essential part of the substructure that is responsible for maintaining the homeostasis within the neuronal environment. Microglia are ubiquitous in the central nervous system (CNS) and, with increasing senescence, their cellular functions deteriorate [26]. It has been observed that the physiological decline in microglia in proportion to the age of the brain in humans is similar to the decline in function in AD. However, these changes are more pronounced in AD patients. This microglia deterioration is accompanied by the release of neurotrophins or inflammatory cytokines which may have detrimental or supportive effect on neurons [27,28,29,30]. In murine models, it has been observed that morphological changes in microglia occurs at a higher rate in aged mice (12 months or older) compared to other age groups [31,32]. Microglia morphology can switch between an active state (i.e., triggered by events such as neuronal death) and a resting or ramified state (i.e., do not phagocytose cells) [33]. In aged mice, these cells are less ramified and more activated, may induce neuronal hypertrophy and become less functional in the CNS. The specific sites of this changes are observed in the corpus callosum, striatum, substantia nigra, dentate gyrus, cerebellar nuclei, inferior cerebellar penducle and the molecular layer of the cerebellar cortex [34,35]. Mouse models that overexpress mutated version of human APP show that these morphological changes in the microglia appear at around six months and are accompanied by a loss of microglial activity in the brain parenchyma [36]. In mice possessing the Familial AD mutation at APP position (PDAPP-JO mice), these changes become observable as early as after three months. In the healthy aged brain, microglia are the main source of pro-inflammatory cytokines such as TNFα, IL1β, IL1α, IL6, nitric oxide, etc. [37,38,39,40]. During AD pathogenesis, they also release IL-4, IL-10, IL-13 and transforming growth factor (TGF)-β1. Studies done on murine models indicate that the increased production of these cytokines may damage neurons in their vicinity, especially since a decrease in TGF-β1 was observed in models that underwent Aβ clearance by phagocytosis during AD pathology [40]. The release of these cytokines and phagocytosis are the main functions of activated microglia; however, researchers have also investigated Ca^2+^ signaling, which may be responsible for microglial regulation. Intracellular Ca^2+^ signaling is responsible for changes in physiological processes such as gene expression, neurotransmitter release, apoptosis and cell differentiation [41]. Any slight change or disturbance in the tightly regulated Ca^2+^ signaling may lead to cellular degeneration. Khachaturian et al. originally asserted that the anomalous regulation of Ca^2+^ in the cell is linked to age-dependent changes that may occur in neuronal function [42]. A recent study in APPPS1 mice as well as in AD patients revealed that the intracellular concentrations of Ca^2+^ are noticeably increased in the activated microglia around Aβ plaques tending towards to the conclusion that the increase in Ca^2+^ signaling is associated with neurodegeneration in AD patients [43,44,45].

Even though data from animal studies suggest that the role of microglia seems to be very relevant with regards to neurodegeneration in AD patients, the problem of interpreting the results of murine models accurately to humans remains a challenge. Studies done on human Alzheimer’s disease tissue (hAD) suggest that Aβ deposits do not directly trigger microglial activation; rather they serve a more passive role since the progressive degeneration and subsequent loss of neuroprotection may contribute towards to the onset of AD cases [29,46]. These findings contradict the mouse model inferences that microglial activation and neuro-inflammation directly contributes towards AD phenotype. Another issue that researchers have contrasting interpretations on is in the case of microglia proliferation. Some studies show that the cellular proliferation may contribute towards AD pathogenesis while others argue against it. For example, it was recently identified that hippocampus of 3xTg-AD mice, when compared to human hippocampus of control subjects, show that microglial proliferation observed in hippocampus of 3xTg-AD was not seen in the human samples [47]. In contrast, another study revealed that there was an increased number of microglia present in the temporal cortex of human AD patients compared to their non-demented control groups [48]. To date the best consensus seems to be that that the phenotypic changes in the microglia are of more importance compared to the increased proliferation rates in AD patients.

## 3. Astrocytes

Several studies have shown that there is a cooperative interplay between astrocytes and neurons that may result in the modulation of cognitive functions [49,50,51,52,53,54]. Several glio-transmitters released from astrocytes may control the synaptic plasticity in the cerebral cortex and hippocampus, and have been shown to be involved in memory and learning. The interruption of astrocyte’s functions, hence in glia transmission, may result in different neuropsychiatric disorders. The neuron–glia interaction may actively control neurotransmission and synaptic plasticity [55,56,57,58,59,60,61]. In the human brain, the neural circuitry is formed by neuronal networks which are embedded in astroglial syncytia. The various functions of astrocytes include neurogenesis, homeostasis, structuring the grey matter, etc. In the early stages of neurodegenerative diseases or traumatic brain injuries, astrocytes often atrophy but remain inactivated; in the later stages, however, they become activated and may cause neuroinflammation that may lead to neurodegeneration and cell death [62]. Studies on hAD tissues and APPswePS1dE9 mice have confirmed that astrocytes in the brain of AD patients upregulate immune response through gene activation and contribute towards chronic inflammation and associated oxidative stress that may cause irrevocable cell damage and ultimate cell death [63,64]. Astrocytes regulate the activity of synapses and partially moderate local blood flow through Ca^2+^-dependent cell signaling [65]. During progression of AD, astrocytes go through noticeable morphological changes. These phenomena are observed in studies of brain tissue of 3xTg-AD mice where astrocytes in the entorhinal cortex show marked atrophy at the early stages, i.e., around one month, well ahead of detectable Aβ plaque formation [66]. In the same mice, after considerable AD progression and the development of extracellular plaques, the atrophy is still observable even when the astrocytes are not in contact with the plaques [67]. However, in terms of number, there is no marked differences in the rate of proliferation or degeneration when AD human brain tissue was compared to healthy control group tissue via post-mortem analysis [68,69,70]. The formation of Aβ plaques in AD patients often results from a lack of Aβ clearance [71]. Astrocytes are responsible for enlisting several enzymes such as neprilysin (NEP), angiotensin-converting enzyme (ACE), endothelin converting enzyme (ECE), insulin degrading enzyme (IDE) and matrix metalloproteinases that degrade Aβ plagues [72,73,74].

Moreover, when astrocytes detect Aβ released by affected neurons, they withdraw their support from all the neurons in the vicinity creating a feed forward loop that results in the acceleration of Aβ plaque formation [75]. Studies done on hAD samples similarly show that, during AD progression, astrocytes are responsible for accumulating Aβ and, when overloaded, form their own Aβ plaques when they undergo lysis [70]. Astrocytes may upregulate the protein known as glial fibrillary acidic protein (GFAP), which regulates immune response in the CNS, especially in neurodegeneration or traumatic brain injury [76,77] Studies in APP/PS1 mice have shown that the deletion of GFAP, which inhibits astrogliosis, showed a marked increase in Aβ plaque formation thereby confirming the role of astrocytes in Aβ degradation [78]. However, it is worth pointing out that sustained proliferation of astrocytes during AD progression has been correlated with an increase of NFTs in human brain tissue [79].

### 3.1. Astrocytic Ca^2+^ Homeostasis in Alzheimer’s Disease

A significant amount of evidence suggests that Ca^2+^ homeostasis is often altered by Aβ. Murine model studies have revealed that astrocytes in Aβ overloaded tissue have increased concentration of Ca^2+^ [80]. In mouse models, these abnormal Ca^2+^ concentrations have been shown to be associated with the increased activation of P2Y_1_ receptors and transient receptor potential channel 4 [81,82]. In 3xTg-AD mice, evidence suggest that Aβ oligomers may disrupt Endoplasmic Reticulum (ER) Ca^2+^ homeostasis in turn inducing ER stress that leads may to astrogliosis [83]. Increased concentration of Ca^2+^ activates phosphatase calcineurin (CaN) and downstream targets, for example, nuclear factor kappa-light-chain-enhancer of activated B cells (NF-κB) and nuclear factor of activated B cells (NFAT). In Tg2576 mice, NFAT or CaN inhibition decreases neuronal degeneration induced by Aβ. In addition, evidence from studies in rat primary astrocytes suggests that CaN activation may contribute towards Ca^2+^ overload [84,85].

### 3.2. Neurotransmitters and Its Involvement with Astrocytes in Alzheimer’s Disease

At the synapse, the clearance of excess glutamate, its conversion to glutamine and the re-synthesis of glutamate are some of the primary astrocytic functions. Glutamate aspartate transporter (GLAST), glutamate transporter—1 (GLT-1) and glutamine synthase (GS) are expressed by these cells for the purpose of maintaining this glutamate homeostasis. In 3xTg-AD mice and hAD samples, GS expression is attenuated by the progressive formation of Aβ [86,87]. In the hippocampus and cortex of AD patients, there is a noticeable decrease in GLT-1 expression which is further evidence of this disruption in glutamate homeostasis [88,89]. This disruption may play a role in glutamatergic transmission and neuronal damage in AD. Moreover, studies on the cerebellum of healthy murine models show that astrocytes release GABA(γ-aminobutyric acid) through bestrophin channel (Best 1) which inhibit synaptic function; GABA is a primary neurotransmitter in the mammalian CNS [88]. From the hippocampus of APP/PS1 mice, it has been observed that there is an enhancement of this Best 1-mediated release of GABA, in the vicinity of Aβ plaques, from active astrocytes indicating that Aβ plaques may play a role in increased astrocytic GABA synthesis. Furthermore, studies on hAD samples show that there is an increased expression of monoaminooxidase B, which is a GABA-synthesizing enzyme in astrocytes, and the total number of GABA positive astrocytes as a whole further clarifying the role of GABA positive astrocytes in AD [90]. All of these findings taken together show strong correlation of astrocytes in the involvement of AD progression.

## 4. Oligodendrocytes

Another important class of glial cells that have been part of AD research are oligodendrocytes. These cells are responsible for providing insulation and support to the neuronal axons in the CNS required for the fast action potential propagation. Evidence suggests that structural and spatial organization of myelin lipid bilayers have a strong connection with the pathogenesis of AD [91,92]. As with microglia and astrocytes, oligodendrocytes have also been shown to exhibit specific morphological changes during AD progression. In 15-month-old PDAPP mice as well as hAD samples, researchers have used imaging studies and X-ray diffraction to show that near the Aβ plaques, there is noticeable deterioration in myelin integrity and axonal destruction [91,93,94,95,96]. Electron microscopy and myelin staining revealed that 2–6 month-old 3xTg-AD mice compared to control groups exhibit myelin sheath alterations and impairment of axonal morphology possibly due to a loss of oligodendrocytes [97,98]. In terms of numbers, however, most studies on mouse models and hAD samples have revealed that, while myelin integrity is compromised in most cases, the overall amount of myelin largely remains unchanged [97,99,100]. When the impact of Aβ is studied on oligodendrocytes, it has been demonstrated through Terminal deoxynucleotidyl transferase dUTP nick end labeling staining and 3-(4,5-Dimethylthiazol-2-YI)-2,5-Diphenyltetrazolium Bromide (MTT) cytotoxicity assay that the treatment with Aβ usually results in the breakdown of cell processes, shrunken cell bodies and DNA damage in these cells [101]. Aβ treatment in oligodendrocytes has also been seen to induce release of cytosolic cytochrome C and increased binding activity of AP-1 and NF-κ suggesting that oxidative stress may be, in part, responsible for cell death [101]. This cytotoxic effect of Aβ on oligodendrocytes was prevented with the introduction of LPS and INF-γ, which are pro-inflammatory in nature, as well as when the cells were co-cultured with astrocytes [102]. However, morphological analysis still showed persistent damage to the oligodendrocytes.

More recently, Aβ treatment on oligodendrocytes have been showed to increase in the levels of caspase-3. The accumulation of caspase-3 leads hindrance of the branching and elongation process of oligodendrocytes by blocking of the local re-organization of microtubules [103]. Park et al. demonstrated that in PS1mutK1 mice, cultured oligodendrocytes exhibit higher levels of Ca^2+^ concentrations compared to control groups which may be a critical factor in ultimate cell death [104]. Another recent study reported that AD progression causes an increase in the DNA damage by upregulating the growth arrest DNA damage protein [105]. Taken together, the changes in functionality and morphology of oligodendrocytes resulting from compromised myelin integrity indicate that oligodendrocytes are also victims during AD progression and eventually contribute towards cognitive dysfunction such as impaired learning ability. However, the extent of involvement of these cells largely remains unknown.

## 5. NG2-Glia

Polydendrocytes or oligodendrocyte precursor cells (OPCs) or NG2-glia are the latest discovery in glial cell types but their role in the pathogenesis in AD are already of major interest [106]. When glial progenitor cells (GPCs), largely composed of NG2-glia, were studied in 12- and 24-month-old APP23 mice, neuroblast percentage in the GPC progeny were markedly reduced compared to control mice [107]. An explanation posited for this phenomenon in GPCs was that there were reduced levels of Mash1, Ngn2 and NeuroD1. In hAD sample, similar observations were seen compared to human control subjects. In addition, the GPC progeny of both models showed reduced levels of β-catenin while glycogen synthase kinase (GSK-3β), an enzyme that phosphorylates β-catenin) and phosphorylated β-catenin levels were increased. In APP23 mice, the expression levels of GSK-3β was reduced accompanied by an increase in β-catenin and Ngn2 when treated with GSK-3β-siRNA. In combination with previous studies, this was evidence of the fact that Aβ activates GSK-3β resulting in the increased phosphorylation of β-catenin resulting in β-catenin degradation and the inhibition of the Wingless/Integrated (Wnt) signaling pathway [108]. This inactivation of the Wnt signaling pathway through Aβ toxicity leads to the inhibition of the differentiation of NG2-glia [109]. However, a recent study also suggests that Aβ does not have a direct impact on the survival rate of oligodendrocytes after the induction of NG2-glia in vitro [103]. A comprehensive investigation by Nielsen et al. of NG2-glia and human AD patients recently revealed that individuals with an overexpression of Aβ showed reduced NG2 immunoreactivity, dense cellular bodies and reduced levels in cell lysates even though cell viability remained largely unchanged. Furthermore, the group reported reduced levels of NG2 in cerebrospinal fluid of AD patient even though there was no link with cognitive decline in those cases [110]. In contrast, another group reported that the number of NG2-glia increased in 12–15-month-old APPswe/PS1dE9 mice compared to age-matched control groups. Indeed, due to the lack of knowledge on the issue, the debate on the exact involvement of NG2 glia remains largely uncertain but this avenue of research may be worthy of pursuit as more and more studies are performed.

## 6. Glial-Oriented Potential Therapeutic Targets of Interest

Recent findings may offer approaches that could potentially advance glial-oriented AD therapy. For example, Evidence suggests that the morphological changes of microglia may be reversed by Aβ vaccination which may reduce Aβ deposits overall [36]. In addition to morphology, genetic studies have revealed that variations in genes that responsible for encoding microglial proteins are strongly related to AD pathogenesis. In a recent study with APPSwe/PS1dE9 mice, it was observed that the gene TREM2 (Triggering Receptor Expressed on Myeloid Cells) is upregulated in microglia and becomes more pronounced as AD progresses [111]. Even though originally TREM2 was identified as a risk gene for AD, this study showed that TREM2 may indeed facilitate Aβ phagocytosis and in turn improve patient cognitive functions by slowing down AD progression by increasing neuroprotection. Furthermore, several recent studies involving TREM2-deficient mice show that such deficiency may lead to a marked increase in Aβ-induced pathology [6,24,112,113]. A very comprehensive review of the role of TREM2 in AD has been published recently by Ulrich et al. that inspects the developing literature concerning TREM2 in AD. In this review, there are some insights provided that highlight the broader role of the innate immune system in neurodegenerative disease and is of interest in AD progression in general [6]. Therefore, TREM2 may eventually become a potential therapeutic target in terms of glial cell oriented therapy for AD. In addition, studies done on APP/PS1 mice have shown that the inhibition of CaN/NFAT pathway in astrocytes slows down astrogliosis and consequently improves cognitive functions [114]. In 2012, Furman et al. showed that using adeno-associated virus (AAV) vectors that contained astrocyte-specific Gfa2 promoter they were able to target the hippocampal astrocytes in APP/PS1 mice. The vectors stimulated the expression of VIVIT, which is a peptide that interferes with the calcineurin/NFAT pathway leading to reduced astrocytic activation. When this treatment was run for several months, the mice showed diminished glial activation, lower Aβ levels and improvements in synaptic and cognitive function. These results are promising and warrant further exploration of novel astrocyte-based therapies for AD.

### 6.1. Antioxidants that Might Be Used to Treat Alzheimer’s Disease

Oxidative stress, i.e., lipid peroxidation and protein oxidation, plays an important role in AD with oligodendrocytes and neurons being more susceptible compared to microglia or astrocytes. Neurons are dependent on astrocytes for the supply of glutathione (GSH) precursors; the Aβ-mediated Ca^2+^ disruption in astrocytes has been shown to result in the death of these neurons. Oligodendrocytes also maintain low concentrations of GSH which in conjunction with higher iron concentrations may attenuate the ability of these cells to scavenge for reactive oxygen species (ROS). Therefore, a reasonable link is formed between Aβ-mediated and antioxidant capacity of the CNS. N-acetylcystein has been shown to prevent Aβ-induced cellular death in oligodendrocytes [101] whereas Trolox, which is a vitamin E analog, has been shown to reduces cell death in astrocytes and neurons [81]. In addition, curcumin has been shown to increase concentrations of GSH by increasing the activity of glutamate-cystein ligase (a GSH synthesizing enzyme) potentially offering up higher neuroprotection from AD [115]. However it may be noteworthy to point out that there is no evidence that the alpha-tocopherol form of vitamin E given to people with mild cognitive impairment prevents the progression to dementia, or even that it improves any cognitive functions in the patients [116,117].

### 6.2. Stimulation of Wnt Pathway in the Treatment of Alzheimer’s Disease

Since Aβ action increases the expression levels of GSK-3β and phosphorylated β-catenin, stimulation of the Wnt/β-catenin pathway may be a potential way to mitigate AD pathology [109]. In vitro and in vivo studies, particularly those on 3xTg-AD mice, have shown that GSK-3β inhibitor TWS119 has mitigated impaired myelination of neurons [118]. Similar stimulation of this signaling pathway may have a role in reducing glutamate excitotoxicity in astrocytes as well. In APP/PS1dE9 mice, it was seen that stimulation by pyrrolidine dithiocarbamate (PDTC) increased GLT-1 levels as well as reduced the extent of tau protein phosphorylation which is another major identified component along with Aβ buildup [119]. Furthermore, a recent study on APPswePS1dE9 mice showed that rosiglitazone or lithium caused similar stimulation and reduced astrogliosis, levels of activated microglia and Aβ plaque load [120]. Stimulation of this pathway does come at a cost, however, since studies on hAD samples showed that the increased levels of β-catenin and consequently the increased level of activated microglia leads to an increase in AD neuroinflammation which counteracts the enhanced Aβ clearance through phagocytosis by the same microglia [121].

### 6.3. Role of Polyamines in Alzheimer’s Disease and Potential Treatment Options

A class of biomolecules found abundantly in all living cells and of interest in AD development is Polyamines (PAs). Polyamines are polycations that interact with molecules that are negatively charged, i.e., DNA, RNA, proteins, etc. Polyamines such as putrescine, spermidine and spermine have critical roles in many biochemical pathways, are involved in cell proliferation and differentiation, and have been showed to be involved in major human diseases including AD [122,123]. Polyamines are involved in communication between glial cells and neurons. This communication is more prominent during stress situations such as brain trauma and ischemia. The mechanisms of storage of these PAs are not well known and are subject to further investigation. It is known however that, PAs are involved in the regulation of neuronal and glial receptors and channels in many neurodegenerative diseases. Polyamine precursors such as ornithine, arginine, and proline and PA derivatives such as agmatine, acetyl-spermine, etc. have also been shown to be involved in AD progression. For example, studies performed on autopsied human brain tissue revealed that ornithine decarboxylase, which is a key regulatory enzyme of PA biosynthesis, showed that, in the case of AD, the mean ornithine decarboxylase levels were significantly upregulated in the temporal cortex, lowered in the occipital cortex and un-changed in hippocampus; this was also in line with animal studies. The demonstration that ornithine decarboxylase is involved in mature brain function opens up a way to extrapolate the involvement of abnormal PA system activity in AD [124]. Another PA precursor, L-arginine, is an amino acid which is semi-essential to life and has several metabolites that are bioactive. L-arginine has also been shown to be significantly altered in AD brains tissue. In this study performed by Liu et al., they further illustrated that age-related changes occurred in the tissue concentrations of L-arginine and its metabolites, i.e., L-ornithine, L-citrulline, putrescine, agmatine, spermidine, spermine, γ-aminobutyric acid, glutamate, and glutamine. These changes occurred in a manner that was metabolite and region specific. These findings taken together show that PA metabolism is subject to alteration in AD brains in comparison to normal brain and may be of interest for further study [125,126]. Another recent study done on the PA precursor, i.e., L-ornithine and the enzyme L-ornithine decarboxylase (ODC), which is also involved in the PA system, showed that ODC was significantly overexpressed in some neurons and in AD specific changes in the central nervous system [127].

Polyamines are synthesized by specific enzymes and one of these enzymes, spermidine synthase, is responsible for catalyzing the formation of the spermine precursor, spermidine from the PA putrescine. Cell localization studies performed on murine brain using polyclonal antibodies have revealed that PAs have very limited localization in neurons whereas the most pronounced localization was seen in astrocytes. Moreover, there was some localization observed in oligodendrocytes that maybe a secondary source of extracellular PAs [128]. There is also evidence that retinal glial cells contain K^+^ channel-active PAs. The synthesis of these PAs may be upregulated in pathological conditions and they could modulate glial cell function and proliferation through modulation of K^+^ signaling [129,130]. Studies done on the adult brain tissue, have shown that there is a lack of PA synthesis in neurons and synapses due to limited expression of the necessary enzymes, however, this is not the case in glial cells [131,132]. Under both pathological and non-pathological conditions, studies have indicated that glial syncitium progresses spermidine mainly through connexin (Cx)-based gap junction channel. These findings suggest new roles of PAs in the astroglial network regulation [133,134]. With regards to PAs, a significant difference between glial cells and neurons is that PAs are stored but not synthesized in glial cells, rather they can be released neuronal synaptic activity regulation from these glial cells. It has also been observed that the PAs have low affinity for, but relatively high turnover in, substrates such as poly-specific cation transporters which are regularly expressed in glial cells [135]. Furthermore, PA derivatives of putrescine that were injected axonally in rat sciatic nerves were ultimately seen to be localized in glial cells [136].

The PAs spermidine and spermine are known modulators of glutamate receptors as well as inwardly rectifying K^+^ channels. In general, glial cells provide a support system for neurons by the buffering of extracellular K^+^ and glutamate [137,138,139,140,141]. PAs are responsible for controlling glial Kir channels, more specifically, the dysfunction of a major astrocyte K^+^ channel, Kir4.1, is seen to appear in the early stages of pathogenesis in several neurodegenerative disorders [131,142,143,144]. Studies on A172 human glioblastoma cells and Novikoff cell lines have indicated that, PAs prevent down regulation of Cx43-mediated gap junction communication. This is primarily due to elevated [Ca^2+^] and [H^+^] that typically accompany ischemia and other conditions. The siRNA knockout of Cx43 significantly reduced these gap junctional communication that hint towards the fat that Cx43 gap junctions may be regulated by spermine [133,134,145]. PAs responsible for controlling several cationic channels and important receptors may have an effect on the excitability and Ca^2+^ levels in glial cells and neurons [146,147,148]. These receptors and channels have been shown to be targets for several neurodegenerative diseases including AD [134,148]. Moreover, metabolic profiling of AD brains revealed that there was significant decline in PA concentrations, revealing another aspect of PAs [149,150]. Studies have shown that age-related region-specific decline of PAs in the cerebral cortex is increased by trauma, this directly suggests that PA system dysregulation may contribute to age-related impairments [122,150,151,152,153,154]. Moreover, in the serum of AD patients, it was seen that acetylated spermine, a derivative of spermidine, was downregulated [155,156,157].

In addition, the increase in PA concentrations has been directly correlated with an increase in lifespan by increased cardio and neuroprotection [134,148,158,159,160,161]. Studies done by testing spermidine agonist in age-dependent memory impairment (AMI), have been shown to improve memory in different trauma situations furthering its role in age-dependent processes [162,163]. This fact may be beneficial in treating neurodegenerative disorders for several reasons. One reason may be that spermidine, which is agonist for N-methyl-D-aspartate (NMDA) receptor/channel complex, can modulate its activity, has been shown to be involved in improving memory function in murine models [164,165]. Another is the fact that exogenous PAs have shown to exhibit neuroprotective effects when administered at higher concentrations in animal models. In contrast, it is noteworthy to point out that over accumulation of hydrogen peroxide, which may be released in several stress situations, and PAs may lead to cell death [159,166,167,168]. However, this should not deviate from the fact that PAs may actually have several important roles to play in AD progression [163,169].

Taken together, it would seem that PA function is somewhat controlled by glial cells and this may be proven through a number of studies involving cellular uptake of PAs, PA accumulation in cell and possible PA release through a reverse transport mechanism. PAs released from glial cells may be responsible for regulating neuronal survival by quenching oxidative radicals and aldehydes that facilitate cell death. PAs may rescue biomolecules by making them more resilient to apoptosis and necrosis and this in turn may have implications in AD progression. Spermidine concentration level increase through exogenous administration has been associated with improvement in neuroprotection during human aging, markedly extension of the lifespan of yeast, flies, and worms, and human immune cells and inhibition of oxidative stress in aging mice. In contrast, the depletion of endogenous PAs may lead to hyperacetylation, generation of reactive oxygen species (ROS) and early necrotic death and decreased lifespan [129,133,134,135,148,159,166,167,168,170].

Polyamine targeted therapy may prove to be promising since it has been observed via in vitro spectroscopy and AFM imaging that PAs promote Aβ-peptide fibrillation and regulate Aβ aggregate formation thereby alleviating Aβ induced toxicity [171,172]. In a recent study, spermidine conjugates carrying variants of differently arranged 3,5-dibenzylidenepiperidin-4-one as bioactive motifs were used to target Aβ-peptide aggregates. It was seen that these agents offered up anti-aggregation activity and consequent neuroprotection. Taken together all of these studies invoke an interesting line of thought that speaks to the roles of PAs in AD treatment strategies [173]. Since progressive neurodegeneration is a primary concern when it comes to stress, ischemia or any CNS related disease such as AD, the proper identification of potential neuroprotective mechanisms may be key in uncovering new therapeutic targets [148].

### 6.4. Other Potential Glial-Oriented Strategies

Neural stem cells (NSCs) engraftment holds much promise in AD since NSCs are responsible for producing various beneficial factors such as neurotrophic factors that promote the regeneration of the CNS as well as migrating to sites of injury and differentiating into neural cells. Studies on 3xTg-AD mice show that the engraftment increased the number of oligodendrocytes and astrocytes as well as increased levels of brain derived neurotrophic factor (BDNF) but did not alter Aβ deposition or tau protein phosphorylation [174]. Another interesting line of research has been perused through the production of Cortical GABAergic interneurons from embryonic medial ganglionic eminence (MGE) cells [175,176]. Immature progenitor cortical interneurons are produced, transported and distributed all through the hippocampus and cortex in the brain. Studies have shown that this particular structure can in fact be dissected from rodent embryos and transplanted to adult animals. The transplant recipients showed promise in several neurodegenerative disorders and these cells may become an attractive target for AD in the near future [177,178,179,180,181,182,183,184,185,186,187,188,189,190]. Furthermore, another group of researchers showed that, in 5xFAD mice, the increased expression of BDNF can be induced by sodium phenylbutyrate and can repair synapses improving cognitive function [191]. In 3xTg-AD mice, intentional glial cell derived neurotrophic factor overexpression also showed conservation of memory and learning capability in vivo as well as decreased oxidative stress and cellular death in vitro [192]. A summary of the glial-oriented potential therapeutic agents are outlined in Table 2.

## 7. Conclusions

The attempt to understand AD in all of its complexities yielded mixed results thus far, but as more data become available, more complex and exhaustive approaches may be identified that will eventuate a more concrete understanding in the near-future. At its core, the understanding of the etiology of AD remains an issue. It is very difficult to pinpoint the exact origin of AD and the key players involved. Currently, no concrete therapy exists that can reverse AD progression and researchers may have to adapt complex approaches to treat AD. Many treatment options have been investigated but, in this review, we only outlined the glial-oriented strategies that showed promise, i.e., TWS119, PDTC, etc. Polyamines (PAs) are a class of biomolecules that may be crucial in this case but further studies are warranted in that regard. To get a better understanding, a more global view of AD needs to be assimilated. Many data exist for AD in terms of neuron-oriented research; together with glial-oriented approaches, small pieces of information are coming out of hiding one-by-one. All of this can be taken together to amalgamate a more macroscopic view of AD that may answer some questions that persist. There is still a long way to go in this line of research but the glial-oriented research into AD may open new doors that can help a significant amount of AD patients around the world.

## Figures and Tables

**Table 1:** Some well-studied murine models for Alzheimer’s Disease and observed alterations in specific types of glial cells.

Murine Model	Observed Loss of Neurons	Observed Alteration in Microglia	Observed Alteration in Astrocytes	Observed Alteration in Oligodendrocytes	Observed Alteration in NG2 Glia
PDAPP-J20	✓	✓	✓	✗	✗
Tg2576	✗	✓	✓	✗	✗
APP23	✓	✓	✓	✗	✓
APP NL-F	✓	✗	✓	✗	✗
APPswePS1dE9	✗	✗	✓	✗	✓
5xFAD	✓	✗	✓	✗	✗
3xTg-AD	✓	✓	✓	✓	✗
APP/PS1	✓	✓	✓	✗	✓
PS1mutK	✓	✗	✗	✓	✗

✓ = alterations have been studied and observed. ✗ = alterations have not yet been studied or no observed alterations were reported.

**Table 2:** A summary of the agents that may have therapeutic applications in AD.

Agent	Mechanism of Action
Trolox	Reduced death of neurons and astrocytes [81]
*N*-acetylcystein	Reduced death of oligodendrocytes [101]
Curcumin	Increased concentrations of Glutathione (GSH) in neurons and astrocytes [115]
TWS119	Improved myelination [118]
Pyrrolidine dithiocarbamate (PDTC)	Increased levels of Glutamate Transporter 1 (GLT-1) [119]
Lithium/rosiglitazone	Reduced AB load [120]
Neural Stem Cells (NSCs) (transplantation)	Improved cognitive functions [174]
Sodium phenylbutyrate	Improved cognitive functions [191]
